# From partner synchrony to corner support: interpersonal co-regulation and performance recovery in DanceSport and boxing

**DOI:** 10.3389/fpsyg.2026.1940453

**Published:** 2026-10-07

**Authors:** Nijia Zhang, Kun Yang, Zengjian Liang, Stanislav Sergeevich Gorbachev, Sergey Samuilovich Aganov, Hongda Cui

**Affiliations:** 1 Institute of Physical Education, Sport and Tourism, Peter the Great St. Petersburg Polytechnic University, St. Petersburg, Russia; 2 Department of Boxing, Kickboxing and Eastern Martial Arts named after K. V. Gradopolov, Russian University of Sport “GTSOLIFK”, Moscow, Russia; 3 Department of Philosophy and Social Sciences, Saint Petersburg University of the State Fire Service of EMERCOM of Russia, St. Petersburg, Russia

**Keywords:** boxing, coach-athlete relationship, co-regulation, dancesport, dyadic coordination, ecological dynamics, interpersonal emotion regulation, performance recovery

## Abstract

Sport psychology has often treated performance regulation as predominantly intrapersonal, yet competitive recovery can also be shaped by relational information. This Conceptual Analysis introduces the Relational Co-Regulation and Performance Recovery (RCPR) Model, a perturbation-centered framework for explaining how partner- and coach-generated inputs may alter recovery after within-competition disruption. Crucially, relational regulatory input is defined independently of success: an observable signal is a regulatory attempt because of its timing, content, and plausible task function, not because recovery follows. A co-regulatory transaction occurs when that input enters a temporally linked interaction loop of detection, appraisal, response, and possible updating; its outcome may be beneficial, null, or dysregulatory. The model also separates psychological state restoration, functional task restoration, and subsequent performance recovery rather than treating them as a single outcome. DanceSport and boxing are used as maximum-contrast theoretical exemplars rather than empirically established sport categories. DanceSport foregrounds predominantly continuous, reciprocal, embodied partner regulation during joint action, whereas boxing foregrounds predominantly intermittent and relatively asymmetrical coach-athlete regulation between rounds. Ecological dynamics, joint action, interpersonal emotion regulation, social support, communal coping, and coach-athlete relationship perspectives are integrated while their distinct explanatory roles are retained. Six core falsifiable propositions address incremental validity, regulatory architecture, matching and uptake, dyadic transmission and co-dysregulation, non-linear dosage constraints, and relational-training specificity. The model generates a research agenda based on perturbation-centered time series, representative simulations, quasi-experimental designs, and converging within-person evidence.

## 1 Introduction

Competitive performance is rarely as individual as the scoreboard implies. Even when a result is attributed to one athlete or one pair, performance regulation is distributed across bodies, relationships, rules, and communication channels. Athletes regulate their own emotion, attention, effort, and action plans, but they also receive information that changes those processes. A partner's touch can stabilize timing, a facial expression can amplify alarm, a concise instruction can reorganize attention, and a trusted voice can restore a workable sense of control after an error. These effects can determine whether a perturbation remains local or develops into a cascade of further mistakes.

The intrapersonal emphasis in sport psychology is understandable. Emotion regulation is commonly defined as the processes by which individuals influence which emotions they have, when they have them, and how they experience or express them (Gross, 2015). Applied practice similarly emphasizes self-talk, imagery, breathing, attentional routines, and cognitive reappraisal. These tools are important, but the athlete is not a closed regulatory system. Emotions are expressed, perceived, and deliberately modified between people; interpersonal regulation can improve or worsen another person's affect, motivation, cognition, and behavior (Zaki and Williams, 2013; Niven et al., 2011). Sport is especially suitable for studying such processes because states are behaviorally consequential, time pressure restricts deliberation, and relational actors often share immediate performance goals (Friesen et al., 2013; Tamminen et al., 2024).

A growing literature shows that athletes regulate teammates‘ emotions, receive regulation from others, engage in communal coping with shared stressors, and are influenced by coaches' emotional expression, communication, and relationship quality (Campo et al., 2017; Leprince et al., 2018; Braun and Tamminen, 2019; Davis et al., 2019; Tamminen et al., 2016, 2019, 2021; van Kleef et al., 2019). Joint-action and ecological-dynamics approaches explain how prediction, shared affordances, information constraints, interpersonal coupling, and reciprocal compensation can organize coordinated behavior (Keller et al., 2014; Silva et al., 2013; Araújo and Davids, 2016). Yet these traditions generally answer different questions. Social support concerns resources and expectations; interpersonal emotion regulation often concerns affective intent or outcome; coach-athlete relationship research emphasizes enduring relational quality; joint-action and ecological approaches explain coordination and perception-action coupling. None of these perspectives, considered alone, specifies a perturbation-centered chain from relational input to appraisal and uptake, restoration in distinct domains, and subsequent within-competition performance recovery.

The RCPR Model is therefore not proposed as a new umbrella label for existing relational constructs. Its intended theoretical contribution is a set of constraints on explanation. First, the unit of analysis is an event-bounded recovery episode beginning with a competitive perturbation. Second, regulatory input is defined independently of whether it succeeds, preventing recovery from being built into the predictor. Third, appraisal and uptake are modeled as intervening processes rather than assumed consequences of exposure to a signal. Fourth, psychological state restoration, functional task restoration, and sport-specific performance recovery are separated so that one can occur without the others. Fifth, regulatory architecture is represented along continuous-intermittent and reciprocal-asymmetrical coordinates rather than treated as a fixed property of a sport. These constraints generate falsifiable predictions about timing, direction, fit, non-linear dosage, and failure.

DanceSport and boxing are selected as a deliberate maximum-contrast pair of theoretical exemplars. The purpose is not to compare the two most similar sports. Pair skating would provide a surface-similar paired and judged comparator to DanceSport, whereas basketball provides an example of physically demanding, interactive team regulation with distributed coordination. Those comparators are important for testing generality, but they do not create the same contrast in relational role and temporal access. The present pairing intentionally spans a performer-performer system in which regulation is embedded in ongoing joint action and an athlete-external-regulator system in which concentrated input is available between action periods. This design makes it possible to ask which RCPR mechanisms survive a large change in relational architecture.

The model contains six process components: competitive disruption; state/task disruption; relational regulatory input; appraisal and uptake; psychological and/or functional task restoration; and performance recovery. A parallel negative route captures co-dysregulation. DanceSport and boxing are treated as theoretical exemplars whose predicted architectures require empirical testing rather than as empirically established categories. This Conceptual Analysis draws selectively on interpersonal emotion regulation, social support, communal coping, joint action, ecological dynamics, coach-athlete relationships, resilience, DanceSport, and combat sport. No original or unpublished data are included. Figure 1 presents the revised RCPR process model, Table 1 compares the two exemplar architectures, Table 2 provides a construct glossary and discriminant map, and Table 3 organizes six core propositions by theoretical status, preferred test, disconfirming evidence, and current feasibility.

**Figure 1:**
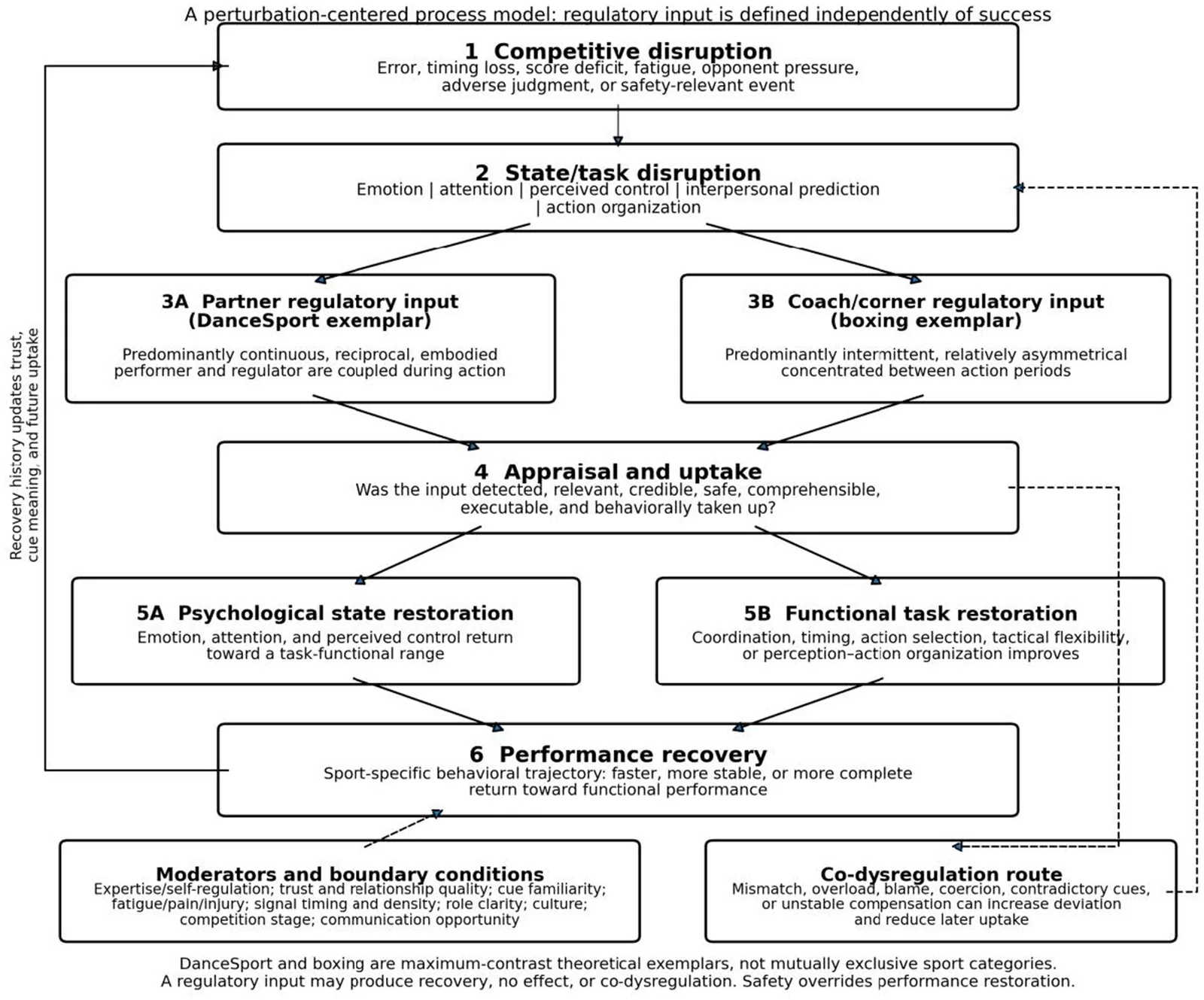
Relational Co-Regulation and Performance Recovery (RCPR) Model. Relational regulatory input is defined independently of success. After a competitive disruption, partner- or coach-generated input may be appraised and taken up, producing psychological state restoration, functional task restoration, both, neither, or a co-dysregulatory trajectory. Performance recovery is a subsequent sport-specific behavioral trajectory. DanceSport and boxing are maximum-contrast theoretical exemplars rather than mutually exclusive sport categories.

**Table 1:** Predominant interpersonal regulatory architectures in the two maximum-contrast theoretical exemplars.

Dimension	DanceSport-predominant exemplar	Boxing-predominant exemplar
Case status	Maximum-contrast theoretical exemplar; architecture requires event-level testing.	Maximum-contrast theoretical exemplar; architecture requires event-level testing.
Focal regulatory relationship	Performer-performer: the couple jointly produces the judged movement.	Athlete-external regulator: boxer performs; coach/corner observes, diagnoses, and communicates.
Temporal access	Predominantly continuous during movement, with additional preparation/review before or after a heat.	Predominantly concentrated in bounded between-round intervals; other access is limited and context-dependent.
Directionality	Highly reciprocal and bidirectional; influence may shift across successive actions.	Relatively asymmetrical and usually coach-to-athlete, while boxer report and responsiveness shape the loop.
Main channels	Haptic pressure, posture, frame stability, rhythm, movement amplitude, gaze, facial expression, breathing, spatial compensation.	Verbal/paraverbal communication, gaze, touch, respiration, tactical instruction, confidence cues, hydration, safety assessment.
Primary regulatory functions	Coordination repair, error absorption, shared-control restoration, preservation of expression, re-establishment of mutual prediction.	Orientation, arousal calibration, information prioritization, tactical reorganization, confidence calibration, safety protection.
Typical perturbations	Timing loss, imbalance, spatial obstruction, missed connection, partner hesitation, unanticipated movement, expressive disruption.	Clean strike, loss of distance control, failure to read a feint, ineffective tactics, warning, fatigue, pain, possible impairment.
Evidence of uptake	Adjusted movement amplitude/force, restored phase relation, stable frame, renewed trajectory.	Orientation to coach, cue recall, plan confirmation, observable implementation in next-round exchanges.
Main failure modes	Blame, panic, ambiguous touch, delayed/excessive compensation, role conflict, reciprocal loss of confidence.	Information overload, contradictory voices, catastrophizing, coercive tone, poor prioritization, misreading athlete state.
Recovery indicators	Latency to restored synchrony, error-probability change, timing/phase stability, movement continuity, expert-coded repair quality.	Next-round tactical adaptation, restored distance control, reduced repetition of the same error, response accuracy, tactical variability.
Adjacent/excluded regulators	Coach interaction before/after heats is an intermittent asymmetrical layer excluded from the focal partner comparison.	Sparring partners and other support staff can add additional regulatory layers outside the focal coach/corner interval.
Key moderators	Partner familiarity, shared cue history, role clarity, trust, discipline/choreography, competition stage, fatigue.	Coach credibility, relationship quality, athlete expertise, cue familiarity, fatigue, pain/injury, bout stage, corner role clarity.

Entries are predicted distributions rather than mutually exclusive categories.

**Table 2:** RCPR construct glossary and discriminant map.

RCPR term	Operational definition	Nearest existing construct	Discriminant point/status
Relational regulatory input	Observable partner/coach behavior whose timing, content, or learned meaning plausibly targets task-relevant state or organization; coded without using outcome.	Interpersonal regulatory attempt; coaching instruction	Predictor is defined independently of success; may be intentional or incidental.
Co-regulatory transaction	Temporally bounded loop in which input is detected/appraised and elicits a recipient response that can shape subsequent signaling or implementation.	Interpersonal regulation; regulatory scaffolding	Requires interdependent participation but not equal influence or successful recovery. One-way instruction alone remains an attempt/intervention.
Co-dysregulation	Interactional trajectory in which signals and responses increase deviation from task-functional ranges or reduce later uptake.	Codysregulation in close-relationship research (Reed et al., 2015)	Not claimed as a novel general construct; RCPR applies it to event-level sport performance and task-functional deviation.
Regulatory architecture	Distribution of regulatory opportunities across temporal access, directionality, modality, and role structure.	Relationship type; team structure	A coordinate description, not a fixed sport category.
Psychological state restoration	Emotion, attention, or perceived control moves toward a range compatible with current task demands.	Emotion regulation; state regulation	Proximal psychological outcome; not equivalent to performance recovery.
Functional task restoration	Coordination, timing, action selection, tactical flexibility, or perception-action organization improves after disruption.	Joint-action/coordination recovery	Task-process outcome; can improve without full emotional normalization.
Performance recovery	Sport-specific behavioral trajectory showing faster, more stable, or more complete return toward functional performance after disruption.	Resilience; final performance	Seconds/minutes and event-level; distinct from winning, final score, or longer-term adaptation.
Shared control	Event-level perception that workable agency remains distributed across self and partner after disruption.	Mutual prediction; joint action; perceived control	Proposed DanceSport mediator; not synonymous with coordination or trust.
Compensation window	Hypothesized functional middle range in which partner compensation contains an error without creating a second perturbation.	Reciprocal compensation in synergies	Novel RCPR event-level nonlinear prediction; not established empirical law.
Sequence principle	Hypothesis that orientation/safety assessment should precede complex tactical content when processing capacity is compromised.	Instructional sequencing; coaching communication	Model-generated, state-dependent sub-hypothesis of matching/uptake.
Information-density threshold	Hypothesized threshold or inverted-U between distinct actionable cues and implementation/recovery.	Cognitive load; information prioritization	Novel RCPR non-linear prediction; optimum should shift with expertise, familiarity, fatigue, and arousal.

**Table 3:** Six core falsifiable propositions derived from the revised RCPR Model, including theoretical status and feasibility of strong testing.

Core proposition	Theoretical status	Preferred test and predicted pattern	Evidence that would weaken/refute	Feasibility
1. Incremental, temporally ordered recovery validity	Core integrative claim	Perturbation-centered multilevel/within-person models controlling pre-input state, disruption severity, skill, self-regulation, resilience, and relationship quality. Input + uptake should predict subsequent recovery trajectory.	No incremental association after prior state/severity/covariates; temporal ordering is absent or reversed.	Tier A-high: observational, within-person, quasi-experimental.
2. Regulatory-architecture differentiation	Core architecture claim	Synchronized interaction data. DanceSport exemplar: more ongoing-action and bidirectional effects; boxing exemplar: more between-round coach-to-athlete effects after opportunity is modeled. Test pair skating, team sport, and within-DanceSport coach layers as out-of-sample cases.	Temporal concentration/directionality do not vary by architecture coordinates, or within-sport variation fully overwhelms predicted patterns.	Tier A-high/moderate: observational and comparative.
3. Matching, appraisal, and uptake	Core mechanism claim with sub-hypotheses	Compare matched vs credible mismatched inputs and model mediation through relevance, credibility, safety, comprehensibility, executability, implementation. State and trust should moderate. Includes sequence and autonomy-urgency sub-hypotheses.	Matched inputs show no advantage; appraisal/implementation do not predict restoration; state/trust do not modify effects.	Tier B-moderate: representative simulations/quasi-experiments.
4. Dyadic transmission and co-dysregulation	Core dynamic claim	Lagged dyadic models controlling each actor's prior state. Deviations should transmit; functional responses attenuate propagation; blame/contradiction/unstable correction should amplify it and reduce later uptake. Shared control is a possible DanceSport mediator.	No cross-partner lagged effects; regulatory responses do not alter propagation; negative transactions are no worse than absence.	Tier A-high/moderate: time series, actor-partner, natural experiments.
5. Non-linear dosage constraints	Novel model-generated prediction	Vary compensation magnitude/predictability in DanceSport and actionable cue density in boxing simulations. Expect middle-range/threshold effects and moderation by familiarity, expertise, fatigue, and arousal.	Effects are consistently linear/monotonic; predictability and state do not shift the optimum.	Tier B-moderate: representative experimental work.
6. Relational-training specificity	Novel intervention prediction	Randomize individual mental-skills, relational recovery training, combined training, and active control; test under representative pressure. Relational training should add benefit where relational information is functionally required.	Only relationship ratings improve; no behavioral recovery advantage; gains transfer equally to tasks without relational information.	Tier C-lower: longitudinal randomized/controlled intervention.

## 2 Conceptual foundations and boundaries

### 2.1 From self-regulation to interpersonal regulation

Self-regulation refers broadly to processes through which a person changes or maintains internal states and behavior in relation to goals. In competition, athletes may reinterpret arousal, redirect attention, regulate breathing, inhibit impulsive responses, or update an action plan. Interpersonal regulation is the broader category in which another person's behavior is intended to, or actually does, alter those processes. The influence may be intentional, as when a coach attempts to orient a boxer, or incidental, as when a dance partner's stiffness changes the other's vigilance. It may target emotion, attention, perceived control, confidence, effort, or action selection.

For conceptual clarity, the RCPR Model distinguishes a relational regulatory input from its outcome. A relational regulatory input is an observable partner- or coach-generated behavior whose timing, content, or learned meaning plausibly targets a task-relevant state or action organization. This definition does not require evidence that the input was helpful. A directive, touch, posture change, expression, silence, or compensatory movement can therefore be coded before its effect is known.

The model reserves the term co-regulatory transaction for a temporally bounded interaction in which regulatory input enters an interdependent loop: one actor emits or withholds a potentially regulatory signal; the recipient detects or fails to detect it, appraises it, and responds behaviorally or physiologically; and that response can in turn maintain, terminate, or alter subsequent signaling. The prefix “co” therefore denotes coupled participation in a regulatory loop, not equality of influence. This criterion is especially important in boxing. A coach's one-way instruction is appropriately described as a coaching intervention or interpersonal regulatory attempt until the athlete's state, appraisal, uptake, or feedback is incorporated. When the boxer's report, responsiveness, implementation, or non-implementation shapes the interaction, the episode becomes co-regulatory even though influence remains relatively asymmetrical.

This distinction also prevents successful recovery from being part of the definition of co-regulation. A co-regulatory transaction can facilitate recovery, have no detectable effect, or contribute to co-dysregulation. RCPR is therefore a process model rather than a success label. The same signal can function differently across athletes and moments because the relational transaction is completed through appraisal, uptake, and task context rather than by signal form alone.

### 2.2 Co-regulation is not synonymous with social support

Social support and relational co-regulation overlap, but they are not interchangeable. Social support may refer to perceived availability, received assistance, emotional care, informational guidance, or tangible resources. It can influence wellbeing through stable relational expectations and ordinary interaction rather than through a discrete response to a performance disturbance (Lakey and Orehek, 2011). A supportive partnership may create conditions for effective co-regulatory transactions by increasing trust, predictability, disclosure, and willingness to accept correction. However, perceived support does not establish that an event-level regulatory transaction occurred.

The RCPR distinction is temporal and functional. Researchers should identify a regulatory input independently of its outcome and then test whether the recipient detected and appraised it, whether behavior changed, and whether subsequent state or task organization improved, deteriorated, or remained unchanged. A coach can be generally supportive yet provide unusable between-round information. A partner can be emotionally close yet destabilize coordination through excessive compensation. Conversely, a concise corrective cue may assist task reorganization without being experienced as warm support. Enduring relational resources and event-level regulatory transactions should therefore be measured separately rather than treated as proxies for one another.

Relational regulation theory remains relevant because it emphasizes that affect, cognition, and action can be regulated through interactions with particular people whose styles fit the recipient (Lakey and Orehek, 2011). RCPR narrows this principle to high-pressure recovery episodes in which regulatory fit must satisfy strict temporal, informational, and task constraints.

### 2.3 Co-regulation, emotional contagion, and co-dysregulation

Emotional contagion concerns convergence produced through exposure to another person's expression or behavior, often without deliberate intent. Co-regulation concerns the structure of a regulatory transaction, not emotional similarity and not guaranteed benefit. If one dancer's alarm spreads to the partner and both become rigid, affect has converged while task regulation has deteriorated. Conversely, effective regulation may involve divergence: a calm partner may remain stable rather than mirror distress, thereby providing information that limits escalation.

The term co-dysregulation is not introduced here as a novel general construct. Work on emotional coregulation in close relationships has already distinguished dynamics that stabilize from dynamics that amplify disturbance (Butler and Randall, 2013; Reed et al., 2015). RCPR adapts this distinction to short, task-specific performance episodes. In this manuscript, co-dysregulation denotes a temporally linked interaction in which signals and recipient responses increase deviation from a task-functional range in emotion, attention, control, or action organization. This performance-specific operationalization differs from using codysregulation as a broad descriptor of maladaptive dyadic emotional dynamics across relationship contexts.

Co-dysregulation can arise through blame, panic, ambiguous touch, contradictory instructions, overcontrol, information overload, or reciprocal loss of confidence. It may also emerge from well-intended behavior. A partner who detects an error may overcompensate, producing an unexpected force or timing change that increases the other's prediction error. A corner may correctly identify several tactical problems but deliver more information than the boxer can implement. The empirical contrast is therefore not “supportive vs. unsupportive people” but the direction of the post-input trajectory after prior state and disruption severity are controlled.

### 2.4 Co-regulation, joint action, ecological dynamics, and embodied interaction

Joint action occurs when two or more people coordinate actions in space and time to produce a shared outcome (Sebanz et al., 2006). Real-time coordination depends on prediction, adaptation, and integration of self-generated and partner-generated information. Rhythmic joint action further requires flexible temporal correction: performers must be sufficiently coupled to coordinate while retaining enough autonomy to avoid reproducing each other's errors (Keller et al., 2014). Interpersonal synchrony can support coordinated performance, yet high synchrony is not universally optimal; functional coordination may include brief divergence, leadership transfer, or asymmetric compensation (Marsh et al., 2009).

Ecological dynamics deepens this account by locating regulation in the performer-environment system. Decisions and coordinated actions emerge under interacting individual, task, environmental, and social constraints rather than being fully prescribed by internal plans (Araújo et al., 2006). In team settings, athletes can become attuned to shared affordances and form temporary synergies in which degrees of freedom are coupled and one component can reciprocally compensate for changes in another (Silva et al., 2013; Araújo and Davids, 2016). Interpersonal coordination in interactive team sport likewise draws on environmental information, prior shared organization, and context-sensitive construction of action (Steiner et al., 2017). These perspectives are especially compatible with RCPR concepts such as perturbation, coupling, compensation, timing, and nonlinear reorganization.

RCPR does not treat relational signals merely as verbal messages imposed on otherwise independent performers. In DanceSport, a pressure change, posture adjustment, or altered movement amplitude can be simultaneously mechanical information, an interpersonal constraint, and a regulatory input. Recovery can therefore emerge through embodied reorganization without a consciously represented instruction. In boxing, a coach's verbal cue can alter which affordances the athlete attends to, but uptake remains constrained by the boxer's embodied possibilities, fatigue, pain, opponent behavior, and the informational structure of the next exchange. This is why an instruction that is semantically clear can still fail functionally.

The added explanatory role of RCPR is perturbation-centered and relational. Ecological dynamics and joint-action theories explain how coordination emerges and reorganizes under constraints; RCPR adds a specific sequence for testing when another person's input enters that reorganization, how it is appraised and taken up, and whether subsequent psychological state, task organization, and performance trajectory change. In DanceSport, synchrony can be both an outcome and a channel of regulation, but synchrony itself is not evidence that a regulatory transaction was beneficial. In boxing, the coach-athlete pair can form a temporarily coupled regulatory system even though only the athlete executes the competitive action.

### 2.5 Co-regulation and communal coping

Communal coping occurs when a stressor is appraised as shared and people combine resources to manage it (Lyons et al., 1998). Sport teams use problem-focused, relationship-focused, and emotion-focused communal strategies when facing shared stressors (Leprince et al., 2018). This perspective is valuable because it shifts language from “my problem” to “our problem” and highlights coordinated coping effort.

RCPR is narrower in timescale and does not require symmetrical ownership of the stressor. A dancer's technical error can become a couple-level coordination problem even if responsibility is individual. A boxer can receive relational regulation from a corner team despite being the person physically exposed to the opponent. Functional interdependence, rather than shared responsibility, is the critical criterion. Communal coping can support RCPR processes by creating shared appraisal and collective intention, but co-regulatory transactions can also occur through rapid embodied or instructional signals without an explicit shared-stressor narrative.

### 2.6 Performance recovery vs. resilience and final performance

Resilience in sport is commonly conceptualized as positive adaptation to adversity, supported by psychological and social protective factors (Fletcher and Sarkar, 2012; Sarkar and Fletcher, 2014). Athlete narratives describe “bouncing back,” and team resilience concerns collective responses to pressure across episodes and time (Galli and Vealey, 2008; Morgan et al., 2013). RCPR uses a more proximal timescale: seconds or minutes following an identifiable within-competition perturbation.

A competitive disruption is an event that displaces the athlete or dyad from a task-functional range. Examples include loss of musical timing, collision, an unanticipated partner response, a scoring deficit, a clean opponent strike, fatigue-related technical deterioration, or an adverse judging decision. The resulting disturbance may occur in emotion, attention, perceived control, interpersonal prediction, or action organization.

The revised model separates three related outcomes. Psychological state restoration refers to movement of emotion, attention, or perceived control toward a range compatible with current task demands. Functional task restoration refers to reorganization of coordination, timing, action selection, tactical flexibility, or other task processes. Performance recovery is the subsequent sport-specific behavioral trajectory showing a faster, more stable, or more complete return toward functional performance relative to the disruption and pre-disruption state. These levels should not be collapsed. A boxer may regain tactical distance while still feeling anxious; a dancer may feel reassured while joint timing remains unstable. Psychological state restoration is therefore not universally necessary for task restoration, and neither subjective improvement nor a single corrected action is sufficient by itself to establish sustained performance recovery.

Recovery can be partial, delayed, unstable, domain-specific, or absent. Final outcome is an imperfect criterion because winning and scores combine recovery with baseline ability, opponent quality, judging, and chance. Dynamic indicators are preferable, such as latency to re-establish timing, slope of error reduction, restoration of tactical variability, reduction in repeated exposure to the same attack, or stability across the next sequence of actions.

## 3 DanceSport and boxing as contrasting relational performance systems

### 3.1 Maximum-contrast case logic, adjacent comparators, and unit of analysis

DanceSport and boxing are used as maximum-contrast theoretical exemplars rather than as matched sports. Both involve high physiological and evaluative pressure, rapid perception-action coupling, limited time for reflection, and consequential interpersonal relationships. The contrast is deliberately constructed around two relational-architecture dimensions: temporal access (continuous vs. intermittent) and directionality (predominantly reciprocal vs. relatively asymmetrical). In DanceSport, the focal relationship is performer-performer and embedded in the judged action. In boxing, the focal relationship is athlete-coach/corner and concentrated outside the active round.

This choice differs from selecting the closest surface comparators. Pair skating is arguably closer to DanceSport because it is also a paired, judged, aesthetic-performance sport requiring joint movement and unison. It would be a valuable test of whether RCPR predictions generalize within a broadly continuous reciprocal architecture. Basketball, by contrast, illustrates distributed multi-person regulation in a physically demanding interactive team sport, where coordination can emerge through shared affordances and team synergies and where external coaching is layered onto player-player interaction (Araújo and Davids, 2016; Steiner et al., 2017). Basketball is therefore an important extension, but its many-to-many network would make it harder to isolate the dyadic perturbation-response sequence used to develop the present model. The current cases were selected to maximize architectural contrast before expanding to intermediate and distributed systems.

The unit of analysis also explains why DanceSport coach interaction is not developed as a parallel case in the present article. Where competition format permits coach contact before or after a heat, that interaction may instantiate an intermittent and relatively asymmetrical architecture similar in some respects to boxing corner communication. We intentionally exclude it from the focal comparison because including both partner and coach regulation within DanceSport would mix two relationship types while the model is being specified. The present DanceSport exemplar therefore concerns in-performance partner regulation; coach input is treated as an adjacent regulatory layer and an explicit target for future within-sport comparison.

A DanceSport couple jointly produces the judged performance. Psychological perspectives on performance enhancement in ballroom dance have also been described (Tremayne and Ballinger, 2008). Each dancer's movement changes the other's available actions, and relational information can be exchanged continuously through contact, posture, force, timing, gaze, expression, and movement trajectory. Research on DanceSport partnerships links relationship quality, expressive ties, and athlete engagement to competitive performance, including actor and partner effects (Liu et al., 2023b, 2024), and partnership measures identify communication, coordination, support, and role-related dimensions as central features of the dyad (Liu et al., 2023a).

In boxing, the coach does not execute the competitive action but can influence interpretation and organization of the next round. The relationship is more explicitly hierarchical and access is temporally restricted. Combat sport requires rapid perception of opponent information, inhibition of unsuitable responses, and tactical adaptation under threat and fatigue (Russo and Ottoboni, 2019; Zhang et al., 2022). Importantly, boxing-specific evidence also documents the content and structure of coach-athlete interaction. Halperin et al. (2016) recorded and coded ringside feedback delivered by amateur boxing coaches between competitive rounds, demonstrating that real-time corner communication varies in attentional focus, autonomy support, and valence. Okada (2013) further showed, through moment-by-moment analysis of boxing practice, how coaches and boxers coordinate verbal and embodied resources to jointly accomplish instruction and action. Microethnographic work in Muay Thai additionally identifies diagnosis, strategy, implementation, affirmation, and consolidation as functions of authentic coach-athlete interaction (Hjortborg et al., 2026). These studies provide convergent but still incomplete support for treating boxing as a theoretical exemplar of intermittent and relatively asymmetrical regulation rather than as an empirically settled architecture.

Table 1 therefore summarizes predicted distributions, not essential sport properties. Some DanceSport partnerships are leader-dominant; some boxers engage in highly reciprocal dialogue. Coaches can sometimes communicate outside the focal interval, and dancers may use planned verbal interaction before or after performance. The model predicts where regulatory opportunities are concentrated, not mutually exclusive categories.

### 3.2 Continuous reciprocal co-regulation in DanceSport

DanceSport regulation is predicted to be predominantly continuous because information exchange occurs throughout movement and predominantly reciprocal because each dancer simultaneously influences and responds to the other. Haptic pressure, frame stability, body tone, step timing, movement amplitude, gaze, facial expression, and breathing can all become regulatory inputs. Their meaning is often inseparable from technical execution: a firmer frame can be both a biomechanical adjustment and information about intended stabilization.

This architecture provides high temporal resolution but little time for explicit explanation. Regulatory success is likely to depend on shared sensorimotor history. Familiar partners can learn the meaning of small changes and distinguish intentional correction from noise. Partnering changes joint movement range and step characteristics, demonstrating that performance possibilities are relational rather than the sum of two independent movement profiles (Yoshida et al., 2020). From an ecological perspective, compact embodied cues can function as informational constraints that reorganize the coupled system rather than as verbal instructions translated into action.

Continuous access also creates vulnerability. Competitive ballroom dance elicits social-evaluative stress responses (Rohleder et al., 2007), and anxiety or uncertainty can be transmitted through tension, delayed response, grip changes, disrupted rhythm, or visible expression. Because each dancer is both regulator and regulated, disturbances can form rapid feedback loops. The dyad may recover through reciprocal compensation or deteriorate through mutual overcorrection. These patterns remain hypotheses about event-level regulation and should not be inferred solely from general partnership quality.

The DanceSport coach is not assumed to be irrelevant. Coach feedback before or after a heat may affect appraisal, confidence, or tactical planning. It is excluded here only to preserve a clear focal comparison between performer-performer regulation during action and external coach-athlete regulation between action periods. Future studies could compare partner and coach inputs within the same DanceSport competition to determine whether architecture predicts recovery more strongly than sport identity.

### 3.3 Intermittent asymmetrical co-regulation in boxing

Boxing regulation is predicted to be predominantly intermittent because the most concentrated coach-athlete exchange occurs during bounded rest intervals and relatively asymmetrical because the coach typically has greater responsibility for observation, diagnosis, and instruction while the boxer retains responsibility for appraisal and implementation. The corner can also manage breathing, hydration, injury, and safety, so relational input can be emotional, cognitive, tactical, somatic, and protective. Observational evidence from competitive boxing confirms that coaches deliver concentrated feedback during between-round intervals, although the functional consequences of specific feedback patterns remain to be tested (Halperin et al., 2016).

Temporal restriction changes the problem. A coach cannot gradually negotiate meaning while the boxer is fatigued and under time pressure. Communication must prioritize. The athlete may have reduced working-memory availability, narrowed attention, elevated arousal, pain, or incomplete recall of the preceding round. A technically accurate message can therefore fail if it is too dense, badly sequenced, or inconsistent with what the athlete perceives as executable.

Relative asymmetry does not imply passivity. The boxer provides embodied and verbal information through report, eye contact, posture, respiration, responsiveness, and subsequent behavior. High-quality coach-athlete relationships are characterized by closeness, commitment, complementarity, and co-orientation (Jowett and Ntoumanis, 2004; Jowett, 2017), and communication strategies help maintain relationship quality and athlete satisfaction (Davis et al., 2019). RCPR predicts that these relational conditions influence whether a coaching intervention becomes an effective co-regulatory transaction through uptake.

The evidence base is less sport-specific than in the DanceSport example. Some claims draw from broader combat-sport perceptual-cognitive research and from Muay Thai coaching rather than boxing-specific process studies (Russo and Ottoboni, 2019; Hjortborg et al., 2026). Boxing should therefore be read as a theoretical exemplar of an intermittent, relatively asymmetrical architecture whose proposed mechanisms require direct empirical testing.

## 4 The relational co-regulation and performance recovery model

Figure 1 presents the revised RCPR Model. The model preserves a perturbation-centered sequence while separating relational regulatory input from its effects and separating psychological state restoration from functional task restoration. The same process components can occur across sports, but their timing, modality, directionality, and role structure differ.

### 4.1 Competitive disruption

The process begins with a perturbation sufficiently salient to disturb current task organization. Disruptions may be discrete, such as a stumble or clean punch, or cumulative, such as repeated failure to control distance. They may originate within the athlete, relationship, opponent, environment, or evaluation system. Their psychological and behavioral effect depends on meaning. A small timing error can be highly disruptive if interpreted as evidence that partner coordination is failing; a strong opponent attack can be contained if it confirms an anticipated pattern.

Disruption should therefore be operationalized at both event and appraisal levels. Event coding can identify observable occurrences, while immediate or retrospective measures can assess perceived severity, controllability, responsibility, and consequence. The model does not assume that every error requires interpersonal regulation. Skilled performers may self-correct automatically. Relational input becomes most consequential when self-regulation is insufficient, when another person's action is part of the correction, or when external information is needed to reorient the athlete.

### 4.2 Athlete or dyad state/task disruption

A disruption can disturb several partially separable domains. Emotional disruption includes escalating fear, anger, shame, frustration, or loss of confidence. Attentional disruption includes fixation on the error, narrowed or scattered selection, and loss of task priorities. Control disruption includes reduced perceived agency, uncertainty about what can be influenced, and conflict over responsibility. Action disruption includes timing instability, rigid repetition, impulsive choice, or breakdown of a shared plan. Interpersonal prediction can also be disrupted when a partner's or opponent's next action becomes less predictable.

These domains interact, but they should not be collapsed into a single generic stress state. Anger can narrow tactical options; uncertainty can increase explicit monitoring; a failed movement correction can further reduce confidence. The regulatory input may need to target the dominant constraint. Reassurance may be useful when relational threat is primary but insufficient when the athlete lacks a clear action rule. Technical instruction may be useful when the plan is unclear but difficult to process under severe arousal or fatigue.

In DanceSport, disruption can exist at individual and dyadic levels. One dancer may remain composed while the couple's timing is unstable. In boxing, the coach can also become dysregulated; a panicked or frustrated corner may transmit urgency without useful prioritization. The model therefore treats both regulator and recipient as state-dependent components of an interactional system.

### 4.3 Relational regulatory input

A relational regulatory input is any observable partner- or coach-generated behavior whose timing, content, or learned meaning plausibly targets a task-relevant state or organization. The input is coded independently of outcome. Intent is informative but not required: an unintentional posture change can alter another performer's organization, while an intentional instruction can fail to be noticed or implemented.

Inputs can serve several functions: stabilization (modulating excessive arousal or volatility); orientation (redirecting attention toward a diagnostic cue or immediate priority); control restoration (highlighting controllable elements); action specification (providing a simple next action or tactical adjustment); confidence calibration (communicating credible belief without denying risk); coordination repair (restoring shared timing, role clarity, or mutual prediction); and safety protection (recognizing injury, cognitive compromise, or conditions in which performance should not continue). These functions are descriptive targets rather than separate latent constructs; one behavior can serve multiple functions.

Signal characteristics include modality, intensity, duration, information density, timing, specificity, consistency, and relational tone. In DanceSport, potentially regulatory inputs are often low-latency and embodied. In boxing, they are more often verbal or paraverbal and compressed into the rest interval. Across both settings, excessive intensity can become noise, and multiple regulators can create conflict when signals are inconsistent. Because outcome is not part of the input definition, identical coded inputs can be compared across episodes that end in recovery, no change, or deterioration.

### 4.4 Appraisal and uptake

Regulatory input does not directly imply recovery. It passes through appraisal and uptake. The athlete determines, explicitly or implicitly, whether the input is relevant, credible, safe, understandable, and executable. This stage explains why the same intervention can produce different effects across relationships and moments.

Trust and relationship quality can shape appraisal because athletes are more likely to accept regulation from someone perceived as competent, benevolent, and attuned to their state. Coaches report that relationship and contextual factors influence interpersonal emotion-regulation strategies (Braun and Tamminen, 2019). Psychological safety may permit athletes to disclose confusion, fatigue, or fear without anticipating humiliation, improving the accuracy of regulation (Jowett et al., 2023). Autonomy-supportive coaching can also increase internalization and adaptive motivation, whereas controlling behavior may produce compliance without flexible uptake (Mageau and Vallerand, 2003).

Uptake also depends on state capacity. High arousal, fatigue, pain, or cognitive overload can reduce available processing. Familiarity can compensate by allowing compact cues to activate practiced routines. Preference matters as well: a cue that stabilizes one athlete may irritate another (Lakey and Orehek, 2011). Effective co-regulation is therefore expected to depend partly on learned cue meanings and prior discussion of preferred responses.

Uptake can be behavioral without conscious endorsement. A dancer may automatically adapt to partner pressure. Conversely, verbal agreement does not prove implementation. Research should measure whether attention, movement, or tactical behavior changed in the predicted direction after the input.

### 4.5 Psychological state restoration, functional task restoration, and performance recovery

The revised RCPR Model separates restoration into two proximal outcomes before performance recovery. Psychological state restoration refers to emotion, attention, and perceived control returning toward a range compatible with task demands. The target is not necessarily calmness; activation may be functional. Functional task restoration refers to reorganization of perception-action coupling, coordination, timing, action selection, tactical flexibility, or another sport-specific task process.

These outcomes can occur in parallel, sequentially, or independently. Psychological state may improve without technical correction, and functional task behavior may improve while anxiety remains elevated. Accordingly, neither state restoration nor task restoration should be treated as a universal mediator. The relevant pathway should be specified before analysis and may differ by perturbation type.

Performance recovery is the subsequent sport-specific trajectory toward functional performance. Potential metrics include latency from perturbation to stable coordination, slope of error reduction, return of movement variability to an athlete-specific functional range, change in opponent-response accuracy, restoration of distance control, or improvement across the opening actions after a corner interval. A single successful action is weaker evidence than a stable trajectory across the next relevant sequence.

Recovery can also be fragile: an initial improvement may be followed by relapse. Repeated perturbation-centered trajectories within one event can reveal whether relational inputs become more or less effective as fatigue and pressure accumulate. Recovery history then feeds back into future appraisal; successful transactions can strengthen cue credibility, whereas failed or coercive interactions can reduce later uptake.

### 4.6 Co-dysregulation

The negative route begins when inputs are absent when needed, mismatched, ambiguous, excessive, coercive, or contradictory, or when recipient responses amplify rather than contain disruption. Four task-specific mechanisms are proposed. Affective amplification occurs when panic, anger, or blame spreads through expression. Attentional competition occurs when added cues compete with essential task information. Control conflict occurs when the athlete experiences input as coercive, incomprehensible, or inconsistent with embodied feasibility. Coordination destabilization occurs when compensatory action is too large, too late, or insufficiently predictable.

Co-dysregulation is an outcome trajectory of the interaction, not a personality label and not evidence that the relationship is globally poor. It can be more harmful than no intervention because it consumes resources and updates future expectations. A dancer who anticipates blame may monitor the partner instead of the task. A boxer who expects conflicting corner voices may disengage from later instruction. The model therefore predicts both immediate disruption and carry-over effects on later signal appraisal and uptake.

## 5 Sport-specific recovery pathways

### 5.1 DanceSport: error absorption, shared control, and relational timing

DanceSport perturbations include timing loss, spatial obstruction, imbalance, missed connection, unanticipated choreography, partner hesitation, and visible expressive disruption. Because the couple jointly produces the performance, the first task after a disturbance is often to prevent local error from becoming mechanically or visibly larger. A partner may maintain musical timing, reduce movement amplitude, stabilize connection, or select a less demanding transition.

The compensation-window hypothesis is explicitly a novel, model-generated prediction rather than an established empirical law. RCPR predicts that the relation between compensation magnitude and recovery is non-linear. Below a minimum, the partner may provide insufficient information or mechanical support to contain the perturbation. Within a functional middle range, compensation can redistribute error cost and help the dyad re-establish a shared trajectory. Above that range, abrupt force, timing change, or leadership transfer may create a second perturbation. This prediction is compatible with ecological-dynamics accounts of reciprocal compensation in synergies (Araújo and Davids, 2016), but the proposed event-level window and its relation to recovery remain to be tested directly.

Shared control is proposed as a second, more proximal mechanism. Here it means an event-level perception that workable agency remains distributed across self and partner: the performer retains action possibilities while relying on the other's predictable contribution. It is not synonymous with joint action or mutual prediction. Joint action describes coordinated performance; mutual prediction concerns anticipating another's behavior; shared control is the hypothesized perception of usable agency within the coupled system. Stable relational signals may increase shared control after disruption and thereby reduce defensive stiffness or indecision, but this mediational pathway remains a testable hypothesis.

The dyadic structure permits lagged partner effects. One dancer's anxiety, attention, or movement deviation can predict the other's next state and the couple's coordination. DanceSport studies using actor-partner interdependence models show cross-partner associations in relationship variables, engagement, and performance (Liu et al., 2023b, 2024). RCPR extends this logic to high-frequency recovery episodes and predicts that functional responses attenuate propagation whereas blame or unstable compensation increases it.

Potential partner-recovery conventions, such as maintaining expression, using a pre-agreed embodied reset cue, or postponing causal discussion until after the heat, should be treated as intervention hypotheses rather than universal prescriptions. Their value depends on role, style, expertise, safety, and the specific perturbation.

### 5.2 Boxing: settling, prioritization, and tactical reorganization

Boxing perturbations include being hit cleanly, losing distance control, failing to read a feint, receiving a warning, accumulating fatigue, or discovering that the planned strategy is ineffective. Some recovery must occur intrapersonally during the round. The corner interval creates a distinct regulatory opportunity in which external observation can be combined with the boxer's embodied report to reorganize the next round.

The sequence principle is a model-generated hypothesis about state-dependent ordering. When a boxer shows high arousal, confusion, or possible impairment, RCPR predicts that orientation and safety assessment should precede complex tactical content. Establishing responsiveness, confirming the athlete's report, and creating a usable attentional channel may improve later uptake. The principle does not imply that every athlete should be calmed; some may require activation. The prediction concerns diagnostic sequencing and should be compared against alternative sequences in representative simulations.

The information-density threshold is likewise a novel falsifiable prediction rather than an established fact. Too little actionable information may leave uncertainty unresolved, whereas excessive information may create competition among cues. RCPR predicts a threshold or inverted-U relation between distinct actionable propositions and implementation, with the optimum shifting upward with expertise and familiar cue systems and downward with fatigue or high arousal. Density should be quantified in meaningful action units rather than word count alone.

Relational tone also requires calibration. The model predicts that task-directive clarity and interpersonal control are separable. A trusted coach may need to be concise and firm when urgency is high, but humiliation, catastrophizing, or denial of the athlete's embodied information may reduce psychological safety, disclosure, and flexible uptake. This autonomy-urgency calibration is therefore best treated as a boundary condition within the broader matching-and-uptake proposition rather than as a separate core proposition.

Multiple corner members introduce a further risk because tactical, emotional, and medical messages can conflict. RCPR predicts that message consistency and role clarity matter independently of the technical quality of isolated instructions. The practical implication is provisional: communication hierarchies should be tested rather than assumed to be universally optimal.

## 6 Moderators and boundary conditions

The RCPR process is moderated by characteristics of the athlete, relationship, event, and culture. Expertise alters perception, automaticity, and cue compression. Experienced dyads and coach-athlete pairs may use brief signals because training has established shared meaning. Novices may need more explicit communication but have less capacity to process it under pressure. Expertise can also increase selectivity: elite athletes may reject input that conflicts with embodied information.

Relationship quality influences trust, disclosure, and tolerance of correction. Closeness without complementarity may not be sufficient; coordinated roles also matter (Jowett and Ntoumanis, 2004). Familiarity can improve prediction but can also entrench dysfunctional patterns. Previous successful recoveries can increase cue credibility, whereas unresolved conflict can turn neutral inputs into perceived criticism.

Fatigue, pain, arousal, and possible injury constrain uptake. In boxing, safety must override performance recovery when the athlete shows signs of neurological or physical compromise. RCPR is not a rationale for persuading an impaired athlete to continue. In DanceSport, musculoskeletal pain or loss of balance may similarly require protective rather than performance-enhancing action.

Competition stage and consequence matter. Early errors allow more time for repair; late errors may require higher-risk adaptation. Cultural norms influence expectations regarding authority, emotional expression, touch, gender roles, and acceptable directness. Regulatory effectiveness should therefore be tested across contexts rather than universalized from one national or coaching culture.

Role structure is also variable. DanceSport disciplines differ in contact, choreography, and leader-follower organization. Boxing formats differ in round length, competition level, and corner access. The proposed architectures are coordinates—continuous to intermittent and reciprocal to asymmetrical—along which specific interactions can be located. Pair skating, basketball, and other sports may occupy intermediate or multi-person positions rather than forming new mutually exclusive categories.

## 7 Core falsifiable propositions and current testability

The revised RCPR Model is organized around six core propositions rather than 12 co-equal propositions. This reorganization distinguishes core mechanism claims from architecture-specific sub-hypotheses and from novel non-linear or intervention predictions. Table 3 also ranks current testability. Tier A propositions can be addressed relatively directly with high-resolution observational, within-person, or quasi-experimental designs; Tier B propositions usually require representative manipulations that may be feasible in training or simulation; Tier C requires longer-term randomized or controlled intervention evidence. In authentic competition, several propositions are unlikely to be settled by a single decisive experiment because relational inputs are endogenous to athlete state. Converging evidence across perturbation-centered observation, representative manipulation, and within-person replication will often be more credible than one nominally “decisive” test.

Proposition 1-Incremental, temporally ordered recovery validity. After pre-input state, disruption severity, athlete skill, trait resilience, individual self-regulation, and general relationship quality are controlled, event-level relational input and uptake will explain additional variance in the speed, stability, or completeness of performance recovery. The critical prediction is temporal: input must precede the measured change, and the association should weaken substantially when prior state and disruption severity are modeled appropriately.

Proposition 2-Regulatory-architecture differentiation. The distribution of regulatory opportunities will vary systematically along temporal-access and directionality dimensions. The DanceSport exemplar should show a higher proportion of partner transactions embedded in ongoing action and more bidirectional lagged effects; the boxing exemplar should show a greater concentration of coach-to-athlete effects between rounds. These are architecture predictions, not claims that all episodes in either sport conform to one pattern. Pair skating, basketball, and DanceSport coach interactions provide important out-of-sample tests.

Proposition 3-Matching, appraisal, and uptake. Inputs that fit the dominant disruption and are appraised as relevant, credible, safe, comprehensible, and executable will be more likely to produce state or task restoration than equally salient mismatched inputs. Athlete state, trust, cue familiarity, and urgency will moderate this pathway. The boxing sequence principle and autonomy-urgency calibration are sub-hypotheses of this broader mechanism rather than separate core propositions.

Proposition 4-Dyadic transmission and co-dysregulation. In coupled performance systems, one actor's deviation will predict the other's subsequent state or action after prior state is controlled. Functional regulatory responses should attenuate propagation, whereas blame, contradictory signals, panic, or unstable overcorrection should increase subsequent deviation and reduce later uptake. Shared control is a proposed mediator within the DanceSport pathway, not a necessary mechanism in every architecture.

Proposition 5-Non-linear dosage constraints. The effect of relational input will not increase monotonically with magnitude or quantity. In DanceSport, compensation is predicted to have a functional middle range; in boxing, actionable information density is predicted to show a threshold or inverted-U relation with implementation. These are explicitly novel RCPR-generated predictions and therefore have a lower prior evidential status than the general temporal and appraisal claims.

Proposition 6-Relational-training specificity. Training relational recovery routines should improve event-level recovery beyond individual mental-skills training alone when task performance depends on relational information, and combined individual-plus-relational training may show the broadest transfer. This intervention proposition is the least directly established and requires controlled, representative training studies before strong applied recommendations are warranted.

## 8 Research agenda

### 8.1 Measure sequences, not only averages

Testing RCPR requires time-resolved designs. Cross-sectional questionnaires can assess relationship climate and perceived regulation but cannot establish whether an input preceded recovery. Researchers should identify perturbation-centered windows containing pre-disruption baseline, disruption, regulatory input, appraisal/uptake, proximal restoration, and subsequent performance. Repeated events nested within athletes, dyads, coaches, and competitions can be analyzed using multilevel time-series models. In DanceSport, actor-partner interdependence models are useful for separating actor and partner effects (Kenny et al., 2006) but should be extended with lagged and event-level structure.

Outcomes should be pre-specified at the appropriate level. Psychological state restoration may be indexed by brief state reports, gaze allocation, response latency, or other validated state markers. Functional task restoration should use sport-specific coordination or tactical measures. Performance recovery should be a subsequent behavioral trajectory rather than a global self-rating. DanceSport outcomes may include time to restore phase relation, center-of-mass stability, step timing, frame variability, or expert-coded continuity. Boxing outcomes may include opening-exchange tactical change, restoration of distance control, reduction in repeated exposure to the same attack, or judge-independent technical indicators. Subjective recovery can complement behavior but should not replace it.

### 8.2 Capture multiple channels

DanceSport studies should synchronize video, inertial measurement, pressure or force information where feasible, audio/music timing, and brief subjective reports. Behavioral coding can identify gaze, expression, contact change, compensation, and post-error routines. Dyadic psychophysiology may be informative, but physiological synchrony should not be interpreted as beneficial without behavioral context.

Boxing studies should record between-round audio and video, identify speaker turns, code input functions, quantify actionable information density, and link messages to next-round behavior. Athlete state can be estimated through respiration, facial behavior, posture, response latency, and brief post-bout stimulated recall. Safety, consent, and protection of sensitive tactical information are essential.

Microethnography is valuable for discovering how compact cues acquire meaning in authentic practice (Hjortborg et al., 2026). Quantitative coding can then test whether identified functions generalize. Mixed methods are particularly appropriate because the same phrase or embodied cue can have different learned meanings across established relationships.

### 8.3 Manipulate fit and sequence in representative settings

Experimental tests should preserve the information and time constraints of competition. Representative learning design cautions against studying sport behavior in tasks that remove the functional couplings present in performance (Pinder et al., 2011). DanceSport experiments might introduce standardized timing perturbations and compare familiar vs. unfamiliar reset cues or graded compensation. Boxing simulations might manipulate instruction sequence, information density, or cue familiarity during standardized between-round intervals, followed by representative opponent scenarios.

The strongest experiments should manipulate input characteristics rather than harm or impairment. Researchers do not need to create dangerous competitive disruptions in order to test RCPR. State-stratified trials, natural perturbations, video-based replay, controlled sparring scenarios, or training tasks can preserve key informational constraints while respecting safety. Ecological validity should be evaluated explicitly rather than assumed.

Randomized interventions can compare individual skills, relational skills, combined training, and active control conditions. Because the intervention proposition is lower in current evidential maturity, early trials should first establish whether training changes observable regulatory transactions and proximal recovery before claiming downstream performance benefits.

### 8.4 Develop measures without collapsing distinct constructs

A future RCPR measure should distinguish regulatory opportunity, input function, appraisal, uptake, psychological state restoration, functional task restoration, and performance outcome. It should not merely ask whether a partner or coach was supportive. Event-level items could assess whether an input was noticed, understood, trusted, feasible, and followed, while behavioral coding identifies what the athlete actually did next.

Measure development should establish content validity with athletes and coaches, test measurement invariance across gender, expertise, culture, and sport, and relate self-report to behavioral indicators. Continuous embodied and intermittent verbal architectures may require different measurement formats. Network or profile approaches may be more appropriate than a single total score because effective regulation can arise through different combinations of functions.

Table 2 provides provisional discriminant definitions to guide this work. In particular, shared control should be empirically distinguished from mutual prediction and general relationship trust, and task-specific co-dysregulation should be distinguished from broad dyadic emotional codysregulation. Demonstrating discriminant validity is a requirement for the model rather than an assumption of it.

### 8.5 Protect causal inference and rank test feasibility

Relational input is endogenous: more severe disruptions often elicit more or different intervention. Simple associations can therefore make helpful regulation appear harmful or ineffective. Studies should control pre-input state and disruption severity, use within-person comparisons, and where possible manipulate input characteristics rather than the occurrence of harmful events. Intent should be coded separately from effect.

Current propositions differ in the feasibility of strong causal testing. Architecture and dyadic-transmission claims are well suited to synchronized observational data and quasi-experimental contrasts. Matching, sequence, compensation, and information-density claims may be tested more directly in representative training simulations. Competition-level randomized manipulation is often infeasible or ethically inappropriate in combat sport, particularly when it would require inducing impairment, withholding safety-relevant information, or manipulating medically consequential decisions.

Accordingly, several RCPR propositions should be evaluated through converging evidence rather than a single decisive trial. Pre-registered perturbation-centered analyses, micro-randomized or instrumental designs in training where appropriate, natural experiments, repeated within-person replication, and representative simulations can jointly strengthen causal inference. A proposition should be considered supported only when temporal ordering, alternative explanations, and task representativeness converge.

## 9 Practical implications: model-derived hypotheses for practice

### 9.1 DanceSport partner-recovery routines

The practical implications below are hypotheses generated by the model unless direct evidence is cited. RCPR suggests that DanceSport training could treat recovery after common errors as a dyadic skill rather than assuming it will emerge automatically. Couples might identify recurring perturbations, establish one or two low-ambiguity reset cues, and rehearse them under progressively representative pressure. They could also test compensation boundaries: who temporarily stabilizes timing, how movement amplitude is altered, and when ordinary roles resume.

The model further suggests that partners discuss preferred regulatory behavior before competition because identical reassurance or correction can be appraised differently. A no-blame convention—preserving task expression, using an agreed reset, and deferring causal discussion—may be useful for some dyads, but it should be evaluated against alternative routines rather than treated as a universal prescription. Video review can focus on the sequence from perturbation to response and recovery rather than assigning blame for the initial error.

### 9.2 Boxing corner communication

RCPR suggests that corner teams prepare a communication architecture before the bout, but the optimal format remains an empirical question. Potential elements include explicit speaker roles, familiar cue vocabulary, rapid assessment of responsiveness, prioritization of one dominant problem, a small number of executable actions, and confirmation of uptake. The model predicts that such sequencing should be most useful when the athlete is highly dysregulated or overloaded, not that a fixed script should be used in every interval.

The model also predicts a distinction between urgency and hostility. Clear directives may be appropriate, but humiliating, catastrophizing, or contradictory communication may impair disclosure and uptake. Athletes can be asked during training which forms of communication remain usable under fatigue and what signals indicate confusion, injury, or loss of visual clarity. Post-bout review can compare what was said, what was understood, and what was implemented. These recommendations should be treated as testable practice hypotheses until direct boxing-specific evidence accumulates.

### 9.3 Integrated psychological skills

Individual and relational regulation should not be framed as competing approaches. Self-regulation is necessary when interpersonal access is limited and may sometimes be sufficient. Relational regulation can supply information, stability, or task constraints that the athlete cannot generate alone. RCPR therefore predicts that psychological-skills programs may benefit from training both sides of the transaction: emitting clear inputs, diagnosing state, appraising and receiving correction, and returning to autonomous or coordinated execution.

However, the practical claim is conditional. Relational training should be expected to add value primarily where performance depends on relational information. Improvements in perceived relationship quality alone would not establish the predicted mechanism; intervention studies should demonstrate changes in event-level regulatory transactions and behavioral recovery.

## 10 Boundary conditions and limitations

The RCPR Model is intentionally selective. It does not claim that all performance disruptions are psychological or that interpersonal intervention is always needed. Technical limitations, injury, opponent superiority, environmental obstruction, and judging variability can constrain recovery regardless of regulation. Safety overrides performance restoration, particularly when neurological or physical compromise is possible.

DanceSport and boxing are theoretical exemplars, not empirically established sport-specific architectures. The evidential bases are also uneven: DanceSport discussion draws on partnership and coordination research specific to the discipline, whereas the boxing literature includes direct observational work on between-round coaching cues and embodied coach-boxer interaction (Halperin et al., 2016; Okada, 2013) alongside broader combat-sport and Muay Thai evidence. The direct boxing literature is therefore more substantial than implied in the original version, but it remains insufficient to establish the proposed RCPR architecture as a settled sport-specific pattern.

The maximum-contrast design necessarily excludes nearby cases. Pair skating may share many features of continuous reciprocal partner regulation while differing in technical, judging, and risk constraints. Basketball and other team sports introduce distributed multi-person regulation and simultaneous teammate-opponent coupling. These cases could reveal that architecture varies more within than between sports. Likewise, DanceSport coach interaction before or after heats may instantiate an intermittent asymmetrical layer that is intentionally excluded from the focal partner analysis. Future work should compare multiple regulator types within the same sport.

Construct overlap remains a substantive risk. Trust, support, communication quality, co-regulation, coordination, shared control, and co-dysregulation are related. Table 2 provides provisional discriminant definitions, but empirical discriminant validity must be demonstrated. The model will be weakened if its proposed event-level constructs cannot be separated from established measures or if they add no explanatory value beyond existing frameworks.

Causal testing is difficult because regulatory input responds to athlete state. High-resolution observational data are necessary but insufficient, while experimental manipulation can reduce ecological validity or create ethical concerns, especially in combat sport. Some propositions—particularly the compensation window, information-density threshold, and relational-training specificity—are novel model-generated hypotheses with limited direct evidence. Their status has been made explicit rather than presented as established conclusions.

Finally, restoration is multidimensional. Psychological state, functional task organization, and performance trajectory may diverge. No single generic recovery scale is likely to be valid across sports and perturbations. The model should therefore be judged by whether it generates useful, sport-specific, temporally ordered predictions rather than by whether every proposed construct becomes a universal questionnaire dimension.

## 11 Discussion

Sport performance is regulated through relationships as well as within individuals. Existing work on interpersonal emotion regulation, social support, coach-athlete relationships, joint action, and ecological dynamics provides strong reasons to expect interpersonal influence on competitive behavior (Friesen et al., 2013; Jowett, 2017; Tamminen et al., 2024; Araújo and Davids, 2016). The theoretical problem is not whether relationships matter, but how to specify their role after a concrete disruption without redefining successful recovery as “co-regulation.”

The revised RCPR Model addresses that problem by separating four levels that are often collapsed. Enduring relational resources such as trust are moderators; relational regulatory inputs are observable event-level predictors defined without reference to success; appraisal and uptake are transaction mechanisms; psychological state restoration, functional task restoration, and performance recovery are separable outcomes. A coaching instruction can therefore be coded even when ignored, and a co-regulatory transaction can end in improvement, no effect, or co-dysregulation. This non-circular architecture provides a clearer basis for incremental-validity tests against interpersonal regulation, social support, and relationship-quality frameworks.

RCPR also clarifies why “co” can apply to relatively asymmetrical boxing interaction. Co-regulation does not require equal influence; it requires an interdependent regulatory loop. When the boxer reports state, appraises input, implements or rejects it, and thereby shapes subsequent interaction, the system is co-regulatory even if the coach has greater diagnostic authority. If recipient involvement is not observed, the more precise term is regulatory attempt or coaching intervention. This criterion creates a falsifiable boundary rather than assuming all coach communication is co-regulation.

Ecological and dynamical perspectives are not competitors to RCPR. They explain how functional organization emerges from performer-environment coupling, shared affordances, constraints, and reciprocal compensation. RCPR embeds those ideas in a perturbation-centered relational sequence. In DanceSport, regulation can be embodied in the coupled movement itself; in boxing, verbal input can alter attentional and action constraints but remains limited by embodied feasibility. This integration shifts the model away from treating regulation as message transmission and toward context-sensitive reorganization.

The DanceSport-boxing contrast should therefore be interpreted as maximum-contrast theoretical sampling. Pair skating is a critical near-neighbor for the continuous reciprocal architecture; basketball and other team sports provide distributed multi-person extensions; DanceSport coaching provides an intermittent asymmetrical layer within the same sport. If future evidence shows that these architectures vary more within sports than between them, that result would refine rather than invalidate the model's coordinate approach.

Finally, the revised six-proposition structure separates relatively mature integrative claims from novel hypotheses. Temporal ordering, appraisal, uptake, and dyadic transmission can be examined now with high-resolution observational and quasi-experimental methods. The compensation window and information-density threshold are deliberately labeled model-generated predictions, and relational-training specificity requires controlled intervention evidence. The model's practical value should therefore be judged by whether these predictions survive representative tests, not by the plausibility of its recommendations alone.

RCPR is intended to provide a more constrained explanation of when, how, and for whom relational input changes the trajectory from competitive disruption toward renewed functioning. Its strongest contribution will be empirical if future studies can show that perturbation-centered relational information explains recovery beyond self-regulation and established relational constructs, while also identifying clear conditions under which the model fails.
